# Highly Efficient and Comprehensive Identification of Ethyl Methanesulfonate-Induced Mutations in *Nicotiana tabacum* L. by Whole-Genome and Whole-Exome Sequencing

**DOI:** 10.3389/fpls.2021.671598

**Published:** 2021-06-01

**Authors:** Hisashi Udagawa, Hiroyuki Ichida, Takanori Takeuchi, Tomoko Abe, Yoshimitsu Takakura

**Affiliations:** ^1^Leaf Tobacco Research Center, Japan Tobacco Inc., Oyama, Japan; ^2^RIKEN Nishina Center for Accelerator-Based Science, Wako, Japan

**Keywords:** ethyl methanesulfonate, mutation, *Nicotiana tabacum*, whole-exome sequencing, whole-genome sequencing

## Abstract

Tobacco (*Nicotiana tabacum* L.) is a complex allotetraploid species with a large 4.5-Gb genome that carries duplicated gene copies. In this study, we describe the development of a whole-exome sequencing (WES) procedure in tobacco and its application to characterize a test population of ethyl methanesulfonate (EMS)-induced mutations. A probe set covering 50.3-Mb protein coding regions was designed from a reference tobacco genome. The EMS-induced mutations in 19 individual M_2_ lines were analyzed using our mutation analysis pipeline optimized to minimize false positives/negatives. In the target regions, the on-target rate of WES was approximately 75%, and 61,146 mutations were detected in the 19 M_2_ lines. Most of the mutations (98.8%) were single nucleotide variants, and 95.6% of them were C/G to T/A transitions. The number of mutations detected in the target coding sequences by WES was 93.5% of the mutations detected by whole-genome sequencing (WGS). The amount of sequencing data necessary for efficient mutation detection was significantly lower in WES (11.2 Gb), which is only 6.2% of the required amount in WGS (180 Gb). Thus, WES was almost comparable to WGS in performance but is more cost effective. Therefore, the developed target exome sequencing, which could become a fundamental tool in high-throughput mutation identification, renders the genome-wide analysis of tobacco highly efficient.

## Introduction

Tobacco (*Nicotiana tabacum*) is one of the most widely cultivated non-food crops and is now cultivated in more than 125 countries (Kakar et al., 2020). The genus *Nicotiana* contains over 75 naturally occurring species (Knapp et al., 2004). *N. tabacum* is a complex allotetraploid (2*n* = 4*x* = 48) species with a large 4.5-Gb genome that has high repetitive element content (Sierro et al., 2014) that evolved through the interspecific hybridization of the ancestral *Nicotiana sylvestris* (2*n* = 24; maternal donor, S-genome) and *Nicotiana tomentosiformis* (2*n* = 24; paternal donor, T-genome) about 200,000 years ago (Leitch et al., 2008). The two sub-genomes in tetraploid tobacco thus are highly similar to those of its ancestors.

The tobacco plant has long served as a model organism in plant biology and has contributed to the understanding of fundamental biological questions in plant physiology, biochemistry, and genetics. As comprehensive reverse genetic resources, chemically or physically induced mutant libraries are extremely valuable tools for studying gene function as well as for increasing the number of useful alleles in plant breeding (Suprasanna et al., 2015). Mutant libraries have been produced in various crop plants to efficiently generate phenotypic variations, enabling functional analysis of genes and the ability to ameliorate the phenotype in molecular breeding (Gupta et al., 2017; Penna and Jain, 2017; Irshad et al., 2020). Ethyl methanesulfonate (EMS) has been used widely as an effective mutagen for chemical mutagenesis in diverse plant species. In tobacco, EMS mutant libraries were developed to obtain mutants with a range of phenotypes, including reduced alkaloid (Julio et al., 2008; Lewis et al., 2010, 2015) and cadmium contents (Hermand et al., 2014), enhanced leaf yield (Reddy et al., 2012), and disease resistance (Takakura et al., 2018; Udagawa et al., 2020; Zhao et al., 2020). Tobacco carries a pair of duplicated genes (referred to as homeologs) from the S- and T-genomes. Due to this redundancy, loss-of-function mutations in any single homeolog are typically masked by the correspondent, hence limiting the use of forward genetic phenotypic screens. Notwithstanding, such a characteristic allows tobacco to tolerate high densities of induced mutations, they are hidden though functional ones.

TILLING (targeting induced local lesions in genomes) methods have been developed and used to select plants with mutations in genes of interest from mutant libraries (McCallum et al., 2000; Uauy et al., 2009). Conventionally, mutations are detected by PCR or three-dimensional PCR (Reddy et al., 2012) with gene-specific primers followed by analyses of short amplicons using CelI-mediated mismatch cleavage, single-strand conformation polymorphism, or Sanger sequencing. However, the development of massively parallel next-generation sequencing (NGS) has enabled researchers to obtain genomic sequences at lower cost and in a shorter period, boosting whole-genome sequencing (WGS) and assembly of many organisms, including tobacco (Sierro et al., 2014; Edwards et al., 2017). NGS also has been used to select mutants for genes of interest where multiplex PCR is performed using gene-specific primers for many target genes and their sequences are obtained simultaneously, so high-throughput screening is possible (Tsai et al., 2011; Gupta et al., 2017).

Efficient detection of mutations is important for accelerating molecular breeding as well as for functional analysis of mutant genes. If the gene regions of the entire genome of a mutant have already been sequenced, any mutations in the library can be detected without a screening step, thus considerably speeding up reverse genetics and its application in plant breeding. Mutations can be detected more comprehensively by WGS and whole-exome sequencing (WES) using target-capturing panels. For example, in sorghum (*Sorghum bicolor*), mutations were detected by WGS of 256 EMS mutant lines (Jiao et al., 2016) and, in wheat (*Triticum aestivum*), mutations were detected by WES of 2735 EMS mutant lines (Krasileva et al., 2017). All the mutations in these mutant libraries have been cataloged.

Whole-exome sequencing focuses mainly on the genomic regions that encode proteins. Therefore, the detected mutations are more likely to change the phenotypes than mutations in other genomic regions (Kaur and Gaikwad, 2017). Coding sequences are only a fraction of the genome sequence and their proportion depends on the species (Warr et al., 2015). In tobacco, the protein coding regions are only 50.3 Mb (about 1.3% of the entire tobacco cultivar K326 genome), and 73.4% of the genome contains various repeat sequences (Sierro et al., 2014). Coding regions often have lower repeat content in which variations generally have higher genetic impact than in other genomic regions. Therefore, WES is especially effective in detecting mutations in organisms with large genomes and high repeat content, and WES enables deep sequencing of large numbers of samples to identify useful variants for incorporation in molecular breeding strategies. So far, WES has been used to detect mutations in soybean (Bolon et al., 2011), rice (Henry et al., 2014; Ichida et al., 2019), and wheat (King et al., 2015; Krasileva et al., 2017). However, until now, the comprehensiveness of mutation detection by WES compared with that by WGS has not been assessed in plants, whereas it has been done in human (Meynert et al., 2014; Belkadi et al., 2015).

Although NGS has been widely used in a range of organisms for various purposes, the conditions for detecting mutations need to be optimized to remove false positives and false negatives. We developed a set of whole-exon capturing probes for tobacco and tested it by analyzing 19 independent mutant lines produced by EMS mutagenesis. We also investigated the optimum conditions to detect mutations in tobacco. Establishment of the WES technique and efficient bioinformatics analysis enabled comprehensive analysis and rigorous characterization of EMS-induced mutations in tobacco, as well as comparison of the performances of WES and WGS in detecting EMS-induced mutations.

## Materials and Methods

### Plant Material and DNA

Ethyl methanesulfonate-induced tobacco mutant lines (Tajima et al., 2011; Takakura et al., 2018) were used in the present study. Briefly, the seeds of tobacco (*N. tabacum*) cv. ‘Tsukuba 1’ were immersed in either a 0.6% or 0.8% (w/v) EMS solution for 16 h, then rigorously washed with water before grown in a greenhouse. We collected an equal amount of leaf tissue from eight individual M_2_ plants which were reproduced from each of the 1974 M_1_ plants after the mutagenesis. DNA was extracted to create bulked DNA that represented all the induced mutations (Tajima et al., 2011; Takakura et al., 2018). For WES and WGS, we selected 19 lines (NtEMS-01–19) out of the 1974 mutagenized tobacco lines, all of which were Sanger-sequenced for exons 1, 2, and 3 of reference *eIF(iso)4E* genes, as described below.

### Probe Design and Whole-Exome Sequencing

The probe design for exon capture was based on the CDS information for the 41,038 genes defined in the reference genome of tobacco cv. ‘K326’ (Sierro et al., 2014). In addition, six genes defined in the reference genome of a cultivar ‘TN90’ (Sierro et al., 2014) but not in K326 were searched in the K326 genome by aligning them using SPALN (Gotoh, 2008) (Supplementary Table 1). Putative homeologs of two genes defined in the TN90 genome were also added to the design. Because the sequences of these homeologs were present but not defined as genes in TN90, and were not present in K326, we first defined their positions in TN90. The second hit of each sequence in TN90 was defined as the position of the homeolog, then, gene sequences were added to K326 genome and defined (Supplementary Table 2). Repetitive regions 100 bp or greater were masked using RepeatMasker (Smit et al., 2015), WindowMasker (Morgulis et al., 2006b), and DustMasker (Morgulis et al., 2006a). The resulting whole exon sequence set was used to design the probes, which were synthesized using a SureSelect XT Custom kit (Agilent Technologies, Santa Clara, CA, United States). Each probe was 120 bases in length and set for 1× tiling. Following the library preparation and target capturing with the produced custom kit, the resulting exon-enriched libraries were sequenced on a HiSeq 2500 platform (Illumina, San Diego, CA, United States) in paired-end, 2 × 100-bp mode to obtain about 10 Gb sequences per sample.

### Whole-Genome Sequencing

Purified genomic DNA was sheared randomly and converted to sequencing libraries using TruSeq DNA PCR-Free Library Prep Kits (Illumina) according to the manufacturer’s protocol. Paired-end sequencing (2 × 150 bases) was performed using a HiSeq X system (Illumina), using two lanes per sample to obtain about 40× coverage of the tobacco genome (approximately 4.5 Gb).

### Conversion of Target Coordinates to the Latest Genome Release, Nitab-v4.5

The probe (target) locations, which were based on the previous tobacco reference genome release (Sierro et al., 2014), were mapped to the latest release, Nitab-v4.5 (Edwards et al., 2017) for bioinformatics analysis. First, each of the target CDS coordinates was expanded by including 50 bp upstream and downstream of the CDS to produce target sequences. All the target sequences from the same mRNA were combined and used in BLASTN searches to identify the best-match regions in the pseudo-chromosome and unincorporated scaffold sequences in Nitab-v4.5. The NCBI BLASTN program version 2.3.0+ (Camacho et al., 2009) with the ‘–max_target_seqs 1’ option was used to align the target sequences to the Nitab-v4.5 sequences. We extracted the pseudo-chromosome and unincorporated scaffold sequences with 80% or greater similarity to at least one of the combined target sequences. Such unincorporated scaffold sequence joined with 1000 Ns, produced eight pseudo-chromosome-like sequences, designated NtUn1–8. The refined reference sequence, Nitab-v4.5_wes, was produced by combining the pseudo-chromosome sequences (Nt01–24) in Nitab-v4.5 and the NtUn1–8 sequences and used as the reference sequence in the present study. Each target sequence was mapped to the Nitab-v4.5_wes by BLASTN search with the ‘-max_target_seqs 5’ option that considers a maximum of five loci as the transferred location. The alignments with smallest E-value that covered at least 80% of the target sequence were used as their locations in Nitab-v4.5_wes. The annotated genes in the Nitab-v4.5 chromosomes and scaffolds were downloaded in general feature format (GFF) from the Sol Genomics Network website^1^ on December 2019 and used in the present study after converting the coordinates that match the Nitab-v4.5_wes. The resulting Nitab-v4.5_wes sequences, gene definitions, and the transferred target locations are available at zenodo.org (10.5281/zenodo.4393108).

### Bioinformatics Analysis

The WGS and WES reads were mapped to the reference genome sequences (Nitab-v4.5_wes) as described above and mutation detection was conducted using the mutation analysis pipeline implemented on the Hokusai massively parallel computing system as described previously (Ichida et al., 2019). Briefly, the system comprised 840 nodes of Primergy CX2550 M4 computers (two units of Intel Xeon Gold 6148 processors and 96 GB of memory per node; Fujitsu, Kanagawa, Japan) connected by InfiniBand EDR. The clean sequencing reads were mapped to the most likely positions of the Nitab-v4.5_wes using BWA-MEM (Li, 2013), based on the match scores. In case there were multiple locations with an equal score, the program randomly selected one of such locations and marked it as the primary alignment. For mutations, we used GATK version 4.1.2.0 (McKenna et al., 2010) and BcfTools version 1.9 (Li, 2011) with the default parameters. We then filtered the detected mutations as follows. Firstly, mutations shared between two or more mutants were considered background mutations and eliminated, as described previously in Ichida et al., 2019. Limiting the initial filtering to simple background elimination enabled us to determine the appropriate filtering condition for the succeeding process. Second, we eliminated mutations with mutant-type allelic read depths less than 10, after which, also, those with a reported quality score (QUAL) less than 200 in GATK and BcfTools results. PCR and Sanger sequencing of the detected mutations verified the filtering accuracy (as elaborated below and in the “Results” section). To all the variant-calling detections herewith, the same such filtering criteria applied.

### Mutation Verification by PCR and Sanger Sequencing

Detected mutations were verified by Sanger sequencing of PCR-amplified fragments. PCRs were performed with Tks Gflex DNA Polymerase (Takara Bio, Shiga, Japan) and specific primer pairs (Supplementary Table 3) for each mutation. The amplicons were sequenced on a 3730xl DNA Analyzer (Thermo Fisher Scientific). Mutations were verified using ATGC software (GENETYX, Tokyo, Japan). Besides, we obtained Sanger sequence data of the reference genes in 1974 mutagenized tobacco lines to compare the mutation density in the sequenced NtEMS lines. We selected *eIF(iso)4E-S* and *eIF(iso)4E-T* genes as the references because exon 1 of these genes (267 and 251 bp, respectively) have already been sequenced in our previous study (Takakura et al., 2018). We newly sequenced the other exons (exons 2 and 3, 167 and 126 bp, respectively) using the following primer pairs: eIF(iso)4E-S_exon2&3 (5′-CTGGGTTTGTTGTTGTAAAGTA-3′ and 5′-CACAGTTTTC AGTTCAGTAAC-3′) and eIF(iso)4E-T_exon2&3 (5′-CCCCAG TAATGGATTCTACC-3′ and 5′-CAGATACTATTTGACACCA C-3′).

## Results

### Whole-Exome Enrichment by Hybrid Sequence Capturing in Tobacco

We used a commercial target enrichment platform (SureSelect XT Custom kit, Agilent Technologies) and established a whole-exon enrichment method in tobacco. The custom-designed tobacco whole-exome capturing kit contained 517,835 oligonucleotide probes of 120 bases. The probes were expected to capture the 50.3 Mb of coding sequences (CDSs) spanning 41,038 genes that had at least one CDS defined in the reference genome sequence of tobacco cultivar K326 as well as eight manually curated relevant genes in the genome of tobacco cultivar TN90 (Supplementary Tables 1, 2) (Sierro et al., 2014). After we produced the whole-exome capturing probes, a new version of the K326 genome sequence, Nitab-v4.5, which has significantly improved coverage and contiguity, was published (Edwards et al., 2017). Our initial assessment indicated that 37,919 (92.5%) of the 41,046 target genes shared 99% or greater sequence identity and had 80% or greater sequence coverage between the previous (K326) and new (Nitab-v4.5) genome sequences, which indicates that the CDSs were mostly conserved in the two versions of reference sequences. Therefore, we decided to map the target locations to the Nitab-v4.5 sequence and conduct all the analysis using the latest Nitab-v4.5 reference genome sequences. Although the quality of the Nitab-v4.5 genome was a significant improvement over the previous K326 genome, more than one third (1.68 Gb) of the scaffold sequences were still not incorporated into the pseudo-chromosomes. Therefore, we constructed eight chromosome-like sequences (named NtUn1–8) from 9,494 unincorporated scaffold sequences with a total length of 1,226,595,102 bases, which matched with at least one target sequences. The pseudo-chromosome sequences (Nt01–24) were combined with the NtUn1–8 sequences, and the resultant sequence set (named Nitab-v4.5_wes) was used as the reference genome sequence in this study. We mapped the nucleotide sequences of the target regions in the K326 and TN90 genomes to the Nitab-v4.5_wes sequences using BLASTN (Camacho et al., 2009). An *E*-value of less than 1.0 × 10^–10^ was set as the threshold for significant hits, similarly to the one previously used for rice (Ichida et al., 2019). We expected that the physical organization of the genes and CDSs would be largely conserved between the previous K326 genome and new Nitab-v4.5 sequences; therefore, we grouped multiple CDSs from the same and adjacent genes into 41,225 ‘blocks’ that were mapped to the Nitab-v4.5 sequences to minimize the chance of possible misalignments by local similarity. The BLASTN search successfully identified at least one location for all the target genes. Of the 41,046 target genes that were defined in the K326 genome, 39,219 (95.1%) matched single genomic locus in Nitab-v4.5, and the remaining 1990 (4.8%) and 16 (0.04%) matched two and three loci, respectively (Supplementary Table 4). The sequence identity within the target regions was extremely high in the two genome releases; 92.1% of the target gene regions (which included introns and untranslated regions) had sequence coverage greater than 90% and 99.4% of the coding regions shared sequence identity greater than 90% (Supplementary Figure 1). These results indicated that the gene structure and their sequences that were predicted using the previous genome sequences were good enough to be transferred to the new release for better sequence accuracy and contiguity. Thus, we successfully transferred the target coordinates to the latest Nitab-v4.5_wes sequences, and all subsequent analyses were based on the Nitab-v4.5_wes and the converted target definitions.

### Optimization of the Filtering Parameters for Mutation Detection

**FIGURE 1:**
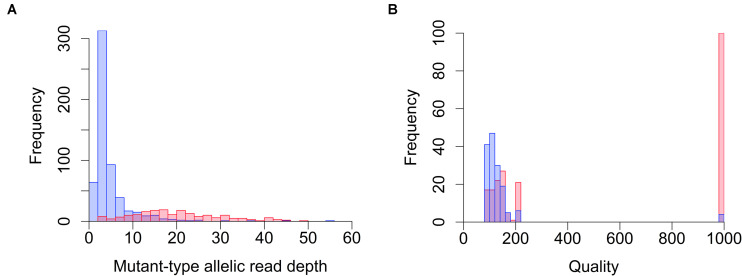
Distribution of allelic read depth and quality scores for visually inspected mutation candidates. Mutation candidates detected by GATK **(A)** and BcfTools **(B)** were inspected using the Integrative Genomics Viewer (IGV). Quality scores are the QUAL values in the VCF outputs. Red and blue bars indicate the frequency of true positive and false positive candidates, respectively.

Whole-genome sequencing and WES often reveal huge numbers of candidate mutations (variants); hence, it is not feasible to verify each mutation individually. Therefore, we first optimized the filtering parameters to effectively remove false positives while retaining real mutations, and to obtain reliable EMS-induced mutations solely by computational methods. Because of its chemical nature, most EMS-induced mutations are C to T transitions (Talebi et al., 2012), therefore mutation detection can be achieved in a relatively straightforward fashion using established methodologies and programs. However, in the present study, mutation detection and filtrating were tricky because all the mutations were expected to be heterozygous due to the pooling of multiple M_2_ plants within a line to reconstruct all the mutations induced in each individual M_1_ plant. Pooling of leaves from multiple plants increased variance in the allelic depth, at least partially, even though we paid extra attention to collect precisely equal amounts of leaf tissue from each plant. We used the WGS results to optimize the filtering condition that is tolerant to bias in the allelic depth of each mutation candidate. The WGS reads of 19 EMS-induced tobacco mutant lines (NtEMS-01–19) and a technical replicate from NtEMS-19 (designated NtEMS-19-rep2; the NGS library independently prepared from the same DNA solution as NtEMS-19) were mapped to the reference Nitab-v4.5_wes sequences, and mutation detection was conducted using the Genome Analysis Tool Kit (GATK) and BcfTools. After removing background mutations shared among two or more of the NtEMS lines analyzed in the same batch, the resulting ‘line-specific’ mutations were chosen randomly, visualized using the Integrative Genomics Viewer (IGV; Robinson et al., 2017) and amplified by PCR followed by Sanger sequencing. We visually inspected 798 candidate mutations that were detected using the GATK on the IGV. We confirmed that 195 (24.4%) mutations existed in the reported locations, whereas 590 (73.9%) resulted in false positives, as there was no mutation at the reported location or the reported variation was presumably due to incorrect mapping between the homeologous genes. During this massive process of visual inspections, we noticed that the mutant-type allelic read depth may be a good indicator of reliable mutations in GATK results. The distribution of mutant-type allelic read depth at the inspected candidate mutations detected by GATK is shown in Figure 1A. Of the 590 candidate mutations that were considered false positive, 519 (88.0%) had mutant-type allelic read depths less than 10, whereas only 27 (13.8%) of the 195 candidate mutations considered true positive had mutant-type allelic read depths less than 10. This result indicated that the HaplotypeCaller tool in GATK requires at least 10 mutant-type reads to produce reliable results in the present condition, and most of the false positives that remained after the line-specificity filtering implemented in our mutation analysis pipeline can easily be eliminated by this simple filtering. As allelic read depths are raw counts of the mapped reads at each locus, it is functional only when enough sequencing reads are available. Besides the minimum read amount required, the above-established criterion is independent of the total read numbers that may differ among the samples. Therefore, it can be applied to both WGS and WES results, if a sufficient amount of read depth is achieved. For BcfTools program, we used the quality scores (QUAL values in VCF outputs) for filtering. The distribution of QUAL values in 362 visually inspected BcfTools results (139 of which were also detected by GATK) is shown in Figure 1B. A clear difference was seen in the distribution of the QUAL values between false positive and true positive candidates; 93.4% (142/152) of the false positive candidates had QUAL values less than 200, whereas 42.4% (89/210) of the true-positive candidates had QUAL values less than 200. Therefore, we decided to exclude mutation candidates that had QUAL values less than 200 as probable false positives. We implemented these criteria in the filtering step of the mutation analysis pipeline and applied them to all the results. GATK and BcfTools detected 6,385,156 and 15,749,156 candidate mutations, respectively, after the removal of background mutations in a total of the 19 independent NtEMS lines and a technical replicate NtEMS-19-rep2. The implemented filtering process by allelic read depth (GATK) and quality score (BcfTools) eliminated 3,824,240 (59.9%) and 10,878,932 (69.1%) mutations as probable false positives, respectively, and left 4,905,173 non-redundant mutations. To evaluate the accuracy of the filtered mutation records, we randomly selected 96 mutation records from the pipeline output (86 mutations were detected by both programs and 10 mutations were detected only by GATK) and verified them by PCR and Sanger sequencing. All 96 mutation records were also detected by PCR and Sanger sequencing, which confirmed the filtering strategy provided sufficient accuracy to detect EMS-induced mutations in tobacco.

### Whole-Exome Sequencing of NtEMS Plants

We aimed to obtain 150× or greater equivalent coverage (about 10 Gb) of the total length of the annotated CDSs (69.9 Mb) in the Nitab-v4.5_wes sequences for each sample. The sequencing statistics are summarized in Supplementary Table 5. The number of read bases varied from 11.6 to 18.9 Gb per sample, which corresponds to 165.3× to 270.8× the total CDS length, which indicates that, as expected, sufficient sequencing data were obtained. More than 98% of the clean bases were mapped to the reference Nitab-v4.5_wes sequences, giving an average read depth of 93.7× (78.3–123.4 in the 19 NtEMS lines and technical replicate) in the CDS regions that commonly existed in Nitab-v4.5_wes and the previous genome releases (target CDSs, 44.8 Mb including 35,456 genes; Supplementary Table 4). An average of 98.7% (98.5%–98.8%), 98.0% (97.8%–98.3%), 97.1% (96.6%–97.7%), and 93.2% (91.4%–95.6%) of the target CDS bases in the 19 NtEMS lines were covered by at least 10, 20, 30, and 50 reads, respectively. Among all the mapped read bases, about 75% (74.9%–77.9%) were located on, or adjacent to, the target regions (a total of 83,696,330 bases; the transferred coordinates of the CDSs and their flanking regions defined in Nitab-v4.5_wes on the basis of the previous genome releases). These results indicated that the whole-exome capturing was successfully done in all the samples, and that there was sufficient coverage of the target sequences for mutation detection.

**FIGURE 2:**
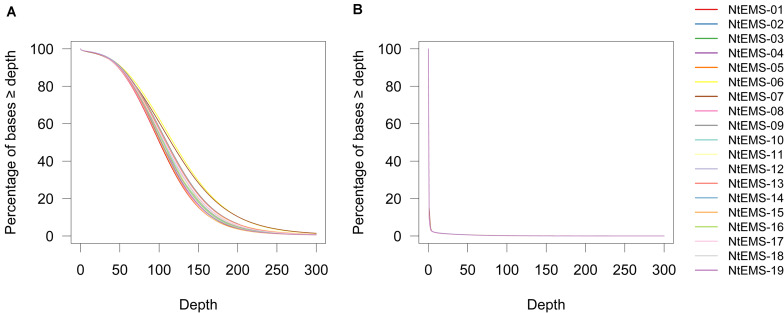
Coverage of 160× whole-exome sequencing in the target coding regions **(A)** and non-target regions **(B)**. Read depths is shown on the *x*-axis.

Next, we down-sampled the sequencing reads to 160× of the total CDS length (69.9 Mb) equivalent, and compared the mapping and variant calling results among the 19 NtEMS lines. The read depth at the defined read volume (160×) gave an average target CDS coverage of 77.2× (72.7–87.8) in the 19 NtEMS lines. An average of 98.6% (98.3%–98.6%), 97.7% (97.4%–97.8%), 96.2% (95.8%–96.4%), and 90.0% (89.1%–91.0%) of the target CDS bases were covered by at least 10, 20, 30, and 50 reads, respectively (Figure 2A). Conversely, an average of 89.1% (84.4%–90.7%) of the non-target sequences (i.e., those outside of the target regions) were not covered by even one read, and only 1.31% (1.25%–1.36%) were covered by 20 or more reads at the 160× target equivalent in the 19 NtEMS lines (Figure 2B). These results clearly show the success of the target enrichment by the designed probes. Because most of the mutations detected in the bulked DNA samples will be heterozygous, the expected mutant allele frequency is 0.5, so 20 reads were considered to be an appropriate threshold to obtain 10 mutant-type reads to accurately assess the covered region.

In the target regions (83.7 Mb, including the 44.8 Mb of the target CDSs that were common between the previous and the current genome releases and their flanking regions), a total of 61,146 mutations were detected in the 19 samples (an average of 3218.2 mutations per sample). As expected, almost all the mutations (99.2%) were judged heterozygous. To assess the technical reproducibility of WES, we compared the detected mutations between NtEMS-19 and NtEMS-19-rep2. Of the 3483 mutations detected in NtEMS-19, 92.7% (3227 mutations) were also found in the technical replicate, NtEMS-19-rep2, demonstrating sufficient reproducibility in two independent target capturing and sequencing reactions. Comparisons between mutations detected in both replicates (“common mutations”) and those found in either one (“replicate-specific mutations”) indicated that between them: (i) there was no significant difference in the read depths (90.5 and 91.5, respectively on average), but (ii) there existed stark contrast in mutant-type allele frequencies (47.2% and 35.5%), indicating that detection of replicate-specific mutations would be particularly challenging if mutant-type allele frequencies are relatively low.

The numbers of detected mutations varied from 1987 to 4966 in the 19 NtEMS lines. The average numbers of detected mutations in each line were 2715.7 (1987–3857) and 3511.3 (2415–4966) in 0.6% and 0.8% EMS treatments, respectively. The differences in the number of mutations between the 0.6% and 0.8% EMS-treated lines were significant (Student’s *t*-test, *p* < 0.05). Therefore, it is likely that the relatively large variation is due to the different EMS concentrations used during the mutagenesis, and the WES was sensitive enough to detect the different mutagenesis efficiency of the different EMS concentrations. We also compared the detected mutations using two different variant calling programs, GATK and BcfTools (Supplementary Figure 2). GATK and BcfTools detected 60,884 (99.6%) and 50,260 (82.2%) of the 61,146 mutations, respectively; 49,998 (81.8%) mutations were common, 10,886 were detected only by GATK, and 262 were detected only by BcfTools. For this reason, the combined use of two programs can achieve a more comprehensive detection of the mutations.

### Property of EMS-Induced Mutations in Tobacco

**FIGURE 3:**
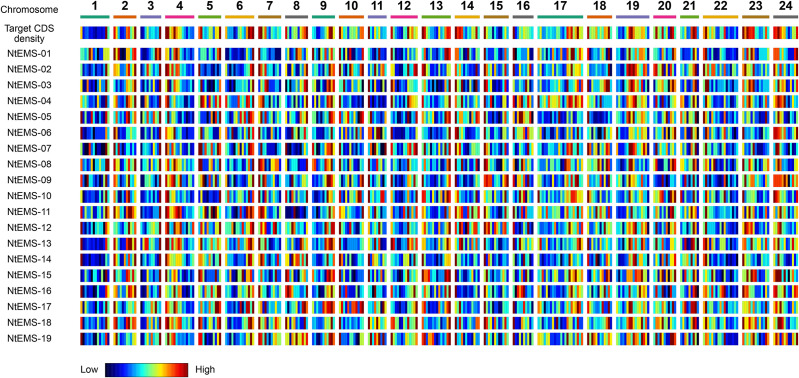
Target and mutation density among the 24 chromosomes in the 19 NtEMS lines. Target coding sequence (CDS) and mutation densities in the 10-Mb genomic segments are shown in the heatmap. The heatmap scale was defined separately for each of the 19 NtEMS lines based on the lowest and highest values in each dataset (horizontal direction), and not globally normalized; therefore, each small bar plot indicates the normalized density in each dataset. The mutation density was largely proportional to the target CDS density in the segment, and had no clear bias.

The EMS-induced mutations in the tobacco mutants were characterized. Almost all (98.8%) of the detected mutations in the 19 NtEMS lines were single-nucleotide variations (SNVs; Supplementary Table 6), and 95.6% of them (57,804/60,439) were C/G to T/A transitions (Supplementary Table 7). This result is consistent with the known properties of EMS mutagenesis (Talebi et al., 2012). Table 1 summarizes the impact of the SNVs on the function of the gene products as predicted by the SnpEff program (Cingolani et al., 2012). A total of 2982 mutations (4.9%; 156.9 mutations per sample, in average) were considered high impact mutations, which are important for the functional analysis of genes of interest. To investigate the genome-wide distribution of the EMS-induced mutations, we calculated the number of mutations detected in a 10-Mb genomic segment in each of the 19 independent NtEMS lines. At 160× of the total CDS length (69.9 Mb) equivalent, the average number of mutations per 10-Mb genomic segment was 7.95 (0–36; Supplementary Table 8). The normalized mutation density, defined as the average number of mutations per 100 kb of target sequences within a 10-Mb genomic segment, was 6.43 (Supplementary Table 9). The mutation density was largely proportional to the target CDS density in the segment and had no clear bias (Figure 3). These results indicated that the EMS-induced mutations were enough random, at least practically, despite base preferences due to the chemical nature of EMS mutagenesis.

**TABLE 1:** Impact of mutations detected in the target regions by whole-exome sequencing on the function of the gene products.

Impact of mutation/effect of HIGH impact^†^	No. of mutations		Rate (%)
	19 samples total	per sample	
HIGH	2,982	156.9	4.9
exon_loss_variant	1		
frameshift_variant	201		
splice_acceptor_variant	410		
splice_donor_variant	412		
start_lost	48		
stop_gained	1,908		
stop_lost	2		
LOW	13,695	720.8	22.4
MODERATE	28,897	1,520.9	47.3
MODIFIER	15,565	819.2	25.5
Total	61,139	3,217.8	100.0

*A summary of the mutations in all 19 independent NtEMS lines is shown in the table; different alleles in different NtEMS lines in the same mutated position were counted as one multiallelic mutation. Therefore, the total number of mutations in this table is different from the number in Supplementary Table 5.^†^Predicted by the SnpEff program (Cingolani et al., 2012).*

Supplementary Figure 3 shows the number of genes with at least one mutation within their target CDS regions. At 160× of the total CDS length equivalent, a total of 20,960 of the 35,667 genes located within the targets harbored at least one mutation in the defined CDS regions in the 19 NtEMS lines by WES. This infers that an average of 1103 genes could be expected to have mutated within their CDS regions in the mutagenized NtEMS population partly analyzed in the present study. Based on the probability formula by Clarke and Carbon (1976), one can expect at least one mutation from 95 lines (at 95% confidence level) in the NtEMS library, for all of the 35,667 genes in the target regions. This result indicated that the high mutation density achieved in the NtEMS mutant population is attractive for the reverse genetics approach in tobacco.

### Comparison Between WES and WGS

We compared the sequence coverage and detected mutations by WES and WGS to determine whether the whole-exon (target) capturing affected the sensitivity and accuracy of variant callings. We obtained an average of 60.7× (52.6–66.4×) genome equivalent of sequencing reads in the 19 NtEMS lines as well as in the technical replicate (NtEMS19-rep2) using the same method used for the WES, except for the target capturing and the recovery. The sequencing statistics are summarized in Supplementary Table 10. In the WGS, an average of 99.3% (98.7%–99.6%) of the target CDSs in the 19 NtEMS lines were covered by at least 20 reads, which is slightly higher than that of the WES coverage (97.7%). A total of 47,259 mutations (an average of 2487.3 and ranged between 1446 and 3818 in each sample) was detected in the target CDSs, whereas a total of 44,202 mutations (an average of 2326.4 and ranged between 1436 and 3588 in each sample) was detected in the 160× WES; that is, 93.5% of the number of mutations detected by WGS were also detected by WES, which indicates that the WES was slightly lower but almost comparable to WGS in comprehensive mutation detection. The mutation frequency calculated from the number of detected mutations and total length (44,832,536 bp) in the target CDSs was 5.19 × 10^–5^ for WES and 5.55 × 10^–5^ for WGS. Sanger sequencing of exons 1, 2, and 3 in reference genes *eIF(iso)4E-S* and *eIF(iso)4E-T* for 1974 tobacco mutants, including the 19 NtEMS lines tested in the present study, detected 113 mutations in 2,179,296 bp as a sum of the sequenced regions. This corresponds to a mutation frequency of 5.19 × 10^–5^, which is similar to the results from the WES and WGS, verifying the consistency of the results obtained by each technique.

### Relationship Between the Amount of Sequencing Data and Sensitivity in Mutation Detection

**FIGURE 4:**
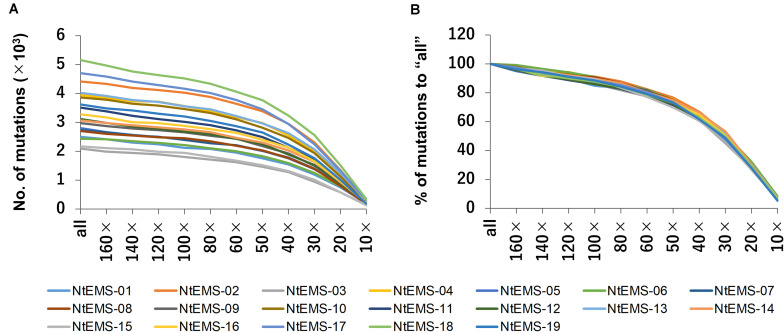
Changes in the number of mutations with different numbers of sequence reads. All the clean sequencing reads (designated as “all”) and down-sampled sequencing reads (160×, 140×, 120×, 100×, 80×, 60×, 50×, 40×, 30×, 20×, and 10× total CDS length equivalent) obtained by whole-exome sequencing were analyzed. **(A)** Number of mutations detected in the target regions. **(B)** Proportion (%) of the number of mutations detected in target regions to “all.”

The effect of differences in the numbers of sequence reads on mutation detection was analyzed using down-sampled sequencing reads (160×, 140×, 120×, 100×, 80×, 60×, 50×, 40×, 30×, 20×, and 10×) as well as all clean sequencing reads before normalization (designated as “all”). Overall, the total number of detected mutations declined gradually as the number of input sequence reads were decreased. For each data set, the numbers of mutations detected in the target regions varied among samples (Figure 4A); however, the proportion of the mutations against “all” was similar (Figure 4B). These results indicate that the proportion of detected mutations was not affected by the number of mutations independently induced in each line, but rather depended on the number of sequence reads.

**FIGURE 5:**
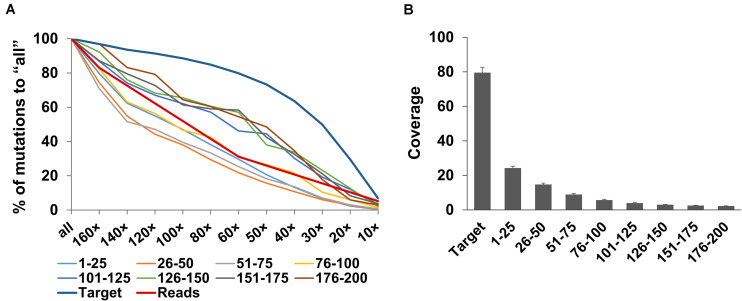
Changes in the number of detected mutations and coverage in the target and flanking regions. **(A)** Percentages of the mutations detected in the target and flanking regions (25-bp bins from the end of the target regions: 1–25 to 176–200) in the down-sampled sequencing reads (160×, 140×, 120×, 100×, 80×, 60×, 50×, 40×, 30×, 20×, and 10× total CDS length equivalent) to those by all the clean sequencing reads (“all”). Red line indicates the change in the percentage of each number of sequencing reads to that of “all.” **(B)** Coverage in the target and the flanking regions at 160× by whole-exome sequencing. Bars indicate the mean and SD in the 19 NtEMS lines.

Strikingly, the rate of decrease in the detected mutations greatly differed between the target regions and outside flanking regions (Supplementary Figure 4 and Figure 5A). In the flanking regions, the numbers of detected mutations decreased as the numbers of sequencing reads decreased (Figure 5A), whereas, in the target regions, the numbers of detected mutations decreased much more slowly as the numbers of sequencing reads decreased. At 160× target equivalent reads, the read depth in the target regions was an average of 79.5 (75.7–88.0), whereas it rapidly decreased as the distance of the flanking regions from the target regions increased; read depth was 24.2 (22.4–26.4) in the flanking regions located 1–25 bp away from each target region and only about 2.1 (1.9–2.4) in the flanking regions located 176–200 bp away (Figure 5B). Given the principle of target capturing, it is reasonable to expect that read depth will be lower in the more distant flanking regions. The differences in the rate of decrease of the detected mutations in the target and flanking regions can be explained by the differences in the read depth: mutations in the target regions had higher read depth, so more mutations had enough read depth to be detected when the amount of sequencing data decreased. In the flanking regions, mutation detection was more sensitive to the amount of sequencing data and many of the mutations had not enough read depth to be detected when the amount of sequencing data decreased.

Whole-exome sequencing requires fewer sequence reads than WGS, which directly reduces sequencing and data storage costs, features that are particularly significant in species like *Nicotiana tabacum* that have large genomes. The analysis of the down-sampled datasets showed that the slope of the proportion of mutations detected in the target regions was gentle when the sequence reads were 50× or more, but the proportion of detected mutations decreased relatively rapidly at 50× or less (Figure 4B). With 60× sequencing reads, the number of reads were an average of about 30% to “all” for the 19 samples, but the detected mutations were 80% to “all.” Therefore, 60× sequencing read would be sufficient to cover a large part of the target genes, when the highest sensitivity was not required.

## Discussion

In the present study, we established a WES procedure that can detect EMS-induced mutations efficiently in *Nicotiana tabacum*, a complex allotetraploid (amphidiploid) with a large 4.5-Gb genome with high repeat content. We first designed 50.3-Mb target regions covering 41,046 genes on the K326 genome release (Sierro et al., 2014), then mapped them to Nitab-v4.5_wes, a modified genome that we constructed based on the latest published K326 genome, Nitab-v4.5 (Edwards et al., 2017). For mutation detection, our mutation analysis pipeline, including both a filter for eliminating inter-cultivar mutations and filtering parameters for two variant calling programs, was optimized to identify mutations correctly by minimizing false positives/negatives. Nineteen individual M_2_ lines were sequenced and mutations on the tobacco genome were analyzed and compared with those detected by WGS.

Whole-exome sequencing is a hybridization-based technology in which the hybridization and analysis are largely dependent on the probe design, the quality of the reference genome, and completeness of gene annotations. It is cost-effective, especially in plant species with large genome, because it can selectively analyze exon regions that generally occupy only 1% − 2% of these genomes. The newly defined Nitab-v4.5_wes genome contained 65,431 genes, 94.1% of the 69,500 genes in the Nitab-v4.5 genome, and the 35,456 genes that were present in both the previous and current K326 genome releases, were selected as the target CDSs in the present study. Most of the 41,046 genes defined in the previous K326 genome release were mapped successfully to the Nitab-v4.5_wes genome in one-to-one correspondence; however, 5590 genes (13.6% of the all genes) that were annotated in the previous genome release were not found in the Nitab-v4.5_wes genome. The regions in the Nitab-v4.5_wes genome that corresponded to these ‘missing’ genes were also subjected to mutation detection. The on-target rate of the WES was about 75%, which is slightly higher but similar to that of rice (72.6%) (Henry et al., 2014), which indicates that the tobacco WES was performed successfully. However, it seems that the probes captured DNA fragments outside the target regions, including flanking regions and the repeat sequences that occupy most of the tobacco genome. The on-target rate could be improved by adding competitive repetitive elements, such as Cot-1 DNA, as blocking DNA to reduce non-specific hybridization, as discussed previously by Ichida et al. (2019) or eliminating repetitive elements from the input library by DSN treatment (Ichida and Abe, 2019).

Ethyl methanesulfonate causes mutations by the alkylation of guanine bases leading to (mis)matches with thymine, which results in transitions of G/C to A/T (Talebi et al., 2012). In the present study, of the 61,146 mutations detected in the 19 NtEMS lines, 98.8% were SNVs (Supplementary Table 6), and as expected, 95.6% of them were C/G to T/A transitions (Supplementary Table 7). Transversions from G/C to C/G or T/A and transitions from A/T to G/C were detected with much less frequency, as has been described in other plants (Minoia et al., 2010; Serrat et al., 2014). The average number of induced mutations with 0.8% EMS was 1.3-fold higher than with 0.6% EMS. In tomato, 1% EMS was reported to yield 1.78-fold more mutations per genome than 0.7% EMS (i.e., one mutation per 322 and 574 kb, respectively; Minoia et al., 2010). Thus, higher EMS concentrations can produce higher mutation densities; however, there is in trade-off between higher mutation densities and embryo lethality and/or seed fertility (Julio et al., 2008; Minoia et al., 2010).

In general, the mutation densities for tetraploid or hexaploid species are higher than those for diploid plant species (Wang et al., 2012). For example, in EMS-induced mutant hexaploid wheat, the mutation density was 10-fold higher than that in EMS-induced mutant diploid barley (Krasileva et al., 2017). The mutation density of the 19 tobacco mutants tested in this study was one mutation per 19.3 kb and 18.0 kb of target regions in the WES and WGS datasets, respectively. These mutation densities are slightly higher than those reported for allotetraploid cotton and hexaploid wheat (one mutation per 26 kb and 24 kb, respectively; Slade et al., 2005; Lian et al., 2020), and more than 10-fold higher than those reported for diploid rice and tomato (one mutation per 294 kb and 367 kb, respectively; Till et al., 2007; Gupta et al., 2017). In allotetraploid tobacco, most genes have two functional copies referred to as homeologs. Because the phenotype by loss-of-function mutations in any single tobacco gene is frequently masked by the gene redundancy, these mutations remain hidden from natural and artificial selection. This is advantageous for the development of mutant populations, because redundancy confers tolerance to deleterious mutations and high densities of induced mutations. The high mutation rates are beneficial for the identification of novel alleles with less screening efforts.

The redesign of the capture probes based on the latest reference genome (Edwards et al., 2017) should be straightforward, and will certainly enhance the coverage in the WES. Because the cost of probe synthesis increases when the target region is enlarged, probe synthesis generally costs more for polyploid plants than for diploid plants. As discussed by Winfield et al. (2012), if probes can be designed to capture both homeologs, the number of probes required to capture the whole exons would be reduced, thus decreasing the cost.

In the WGS and WES, 99.3% and 97.7% of the target CDSs, respectively, were covered by at least 20 reads, and this was enough to detect mutations with good sensitivity and accuracy. The 44,202 mutations detected by WES comprised 93.5% of the 47,259 mutations detected by WGS, which shows that WGS gave slightly better coverage than WES. However, both methods produced comparable results in detecting mutations by EMS treatment. In addition, the amount of sequencing data necessary for efficient mutation detection were significantly lower in WES (11.2 Gb at 160× coverage of the 69.9-Mb target region), which is 6.2% of the amount required in WGS (180 Gb at the 40× equivalent of the 4.5-Gb genome). This demonstrates that WES is cost-effective and practical approach for reverse genetics and its application in breeding. In the present study, there were 4,592,093 mutations located outside the target CDSs and these mutations were mostly only detectable by WGS. It is a reasonable expectation that at least some of the off-target mutations are located on regulatory regions, which may alter the strength or pattern of the gene expressions. Such ‘subtle’ mutants would also be useful for tobacco breeding to achieve a fine-tuning of the phenotypes.

In tobacco, the overall cost for WES (capturing of a total length of ∼50 Mb target regions and 10 Gb of sequencing reads that corresponds to a 200× coverage of the target regions) was roughly 60% of WGS at 40× coverage at the time when we performed these analyzes. Assuming the same total target size, the theoretical genome size to be the break-even point between WES and WGS is 2.7 Gb, which corresponds to 60% of the genome size of tobacco, when target capturing is performed individually. Pre-capture multiplexing in which a single capturing reaction is performed with multiple and mixed samples can also decrease the cost, although it has been reported that pre-capture multiplexing often reduces the capture efficiency (Shearer et al., 2012; Ichida et al., 2019). The effects of this method on the capturing efficiency need to be further investigated in tobacco, especially when analyzing a large population to produce a mutant library ready for reverse-genetics screenings by this method.

Around 80% of the mutations detected in “all” (165.3–270.8× target equivalent) samples were detected at 60× sequencing read coverage in the analysis using down-sampled sequencing reads (Figure 4B). This result indicated that further decreases in the numbers of sequence reads and adjustment of the number of detected mutations may be possible, depending on the purpose. Therefore, the developed target exome sequencing procedure described in the present study will contribute to genome-wide high-throughput mutation identification for comprehensive analysis of mutant populations or for population genetics.

## Data Availability Statement

The original contributions presented in the study are publicly available. The data presented in the study are deposited in the Sequence Read Archive (https://www.ncbi.nlm.nih.gov/sra), accession number PRJNA683986 and Zenodo (https://zenodo.org), Digital Object Identifier 10.5281/zenodo.4393108. All other relevant data are in the manuscript and the Supplementary Material.

## Author Contributions

HU, HI, TA, and YT conceived and designed the experiments. HU and TT performed the experiments. HI designed and performed the bioinformatics analysis. HI and HU analyzed the data. HI, HU, TA, and YT wrote the manuscript. All authors read and approved the final manuscript.

## Conflict of Interest

Part of this project was performed as an internal research activity in Japan Tobacco Inc. HU, TT, and YT are current employees of the company. HI and TA received a research funding for this work from Japan Tobacco Inc., under collaborative research agreement between RIKEN and the company.

## Footnotes

1 ftp://ftp.solgenomics.net/genomes/Nicotiana_tabacum/edwards_et_al_2017/annotation

## Supplementary Material

Supplementary Figure 1 Target coverage and identity in the Nitab-v4.5_wes sequences. Histograms of the BLAST mapping of the target regions to Nitab-v4.5_wes sequences. **(A)** Percentage of query length; **(B)** Percentage identity. Most of the target regions had very high (>90%) coverage and identity.

Supplementary Figure 2 Mutations detected by the variant calling programs GATK and BcfTools. Venn diagram of the mutations detected by GATK and BcfTools. Most mutations were detected by both programs, but some mutations were detected by only one of the programs.

Supplementary Figure 3 The number of possible alternated genes by read amount and the population size required for saturation mutagenesis. Bar graph shows the average number of genes (per line) with at least one mutation within their CDS regions. The number of NtEMS lines required to achieve saturation mutagenesis (right axis) was also shown based on the probability formula by Clarke and Carbon (1976).

Supplementary Figure 4 Stacked bar chart of mutations detected in the target and the flanking regions with different numbers of sequencing reads. The data are the total number of mutations detected in the 19 NtEMS lines with different numbers of sequencing reads: all clean sequencing reads (“all”) and down-sampled sequencing reads (160×, 140×, 120×, 100×, 80×, 60×, 50×, 40×, 30×, 20×, and 10× total CDS length equivalent) by whole-exome sequencing. More mutations were detected in the target regions than in the flanking regions 1–25 to 176–200 distant from the target regions.

Supplementary Table 1 Positions of additional genes that are defined in the genome of tobacco cultivar TN90 but not in K326 (Sierro et al., 2014).

Supplementary Table 2 Positions of unannotated putative homeologs of gene_85817 and gene_74284 in the genome of tobacco cultivar TN90 (Sierro et al., 2014).

Supplementary Table 3 Primers used for the PCR amplifications of selected mutations for verification by Sanger sequencing.

Supplementary Table 4 Translated target (bait) locations in the Nitab-v4.5_wes combined sequence set.

Supplementary Table 5 Whole-exome sequencing (WES) statistics for 19 NtEMS lines and a technical replicate.

Supplementary Table 6 Mutations detected in target regions of the 19 NtEMS lines with 160× whole-exome sequencing data.

Supplementary Table 7 Proportions of the mutations detected in the 19 NtEMS lines that were single-nucleotide variations (SNVs).

Supplementary Table 8 Number of mutations per 10-Mb genomic segments mutations per 10 Mb genomic segment in the target regions on the 24 tobacco chromosomes.

Supplementary Table 9 Number of mutations per 100-kb of target sequences in each 10-Mb genomic segment of the 24 chromosomes.

Supplementary Table 1 Whole-genome sequencing (WGS) statistics of 19 NtEMS lines and a replicate.
