# Supplementary material for: Highly Efficient and Comprehensive Identification of Ethyl Methanesulfonate-Induced Mutations in Nicotiana tabacum L. by Whole-Genome and Whole-Exome Sequencing
Source: Front Plant Sci. 2021 Jun 1;12:671598. doi: 10.3389/fpls.2021.671598 (PMC8204250; doi:10.3389/fpls.2021.671598)
Supplement: Supplementary Figure 1 — Target coverage and identity in the Nitab-v4.5_wes sequences. Histograms of the BLAST mapping of the target regions to Nitab-v4.5_wes sequences. (A) Percentage of query length; (B) Percentage identity. Most of the target regions had very high (>90%) coverage and identity. [file Data_Sheet_1.zip › Supplementary materials/Supplementary Figures.pdf]

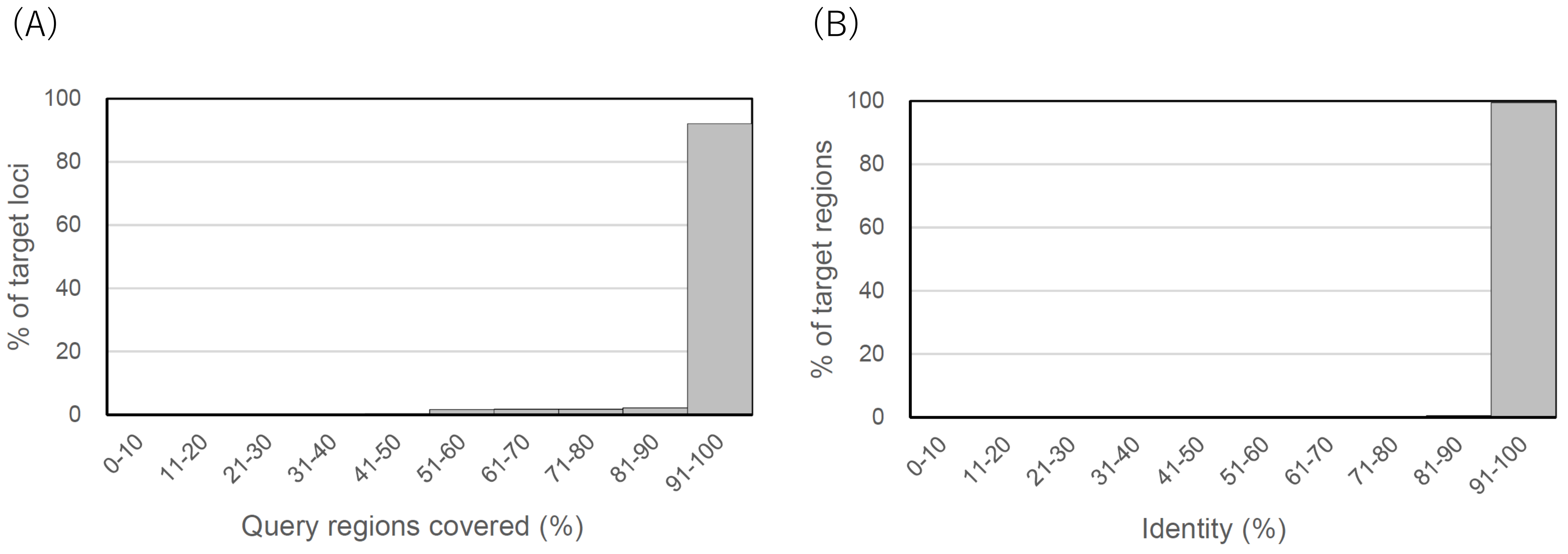

Supplementary Figure 1. Target coverage and identity in the Nitab-v4.5_wes sequences
Histograms of the BLAST mapping of the target regions to Nitab-v4.5_wes sequences. (A) Percentage of query length; (B) Percentage identity. Most of the target regions showed very high (> 90%) coverage and identity.

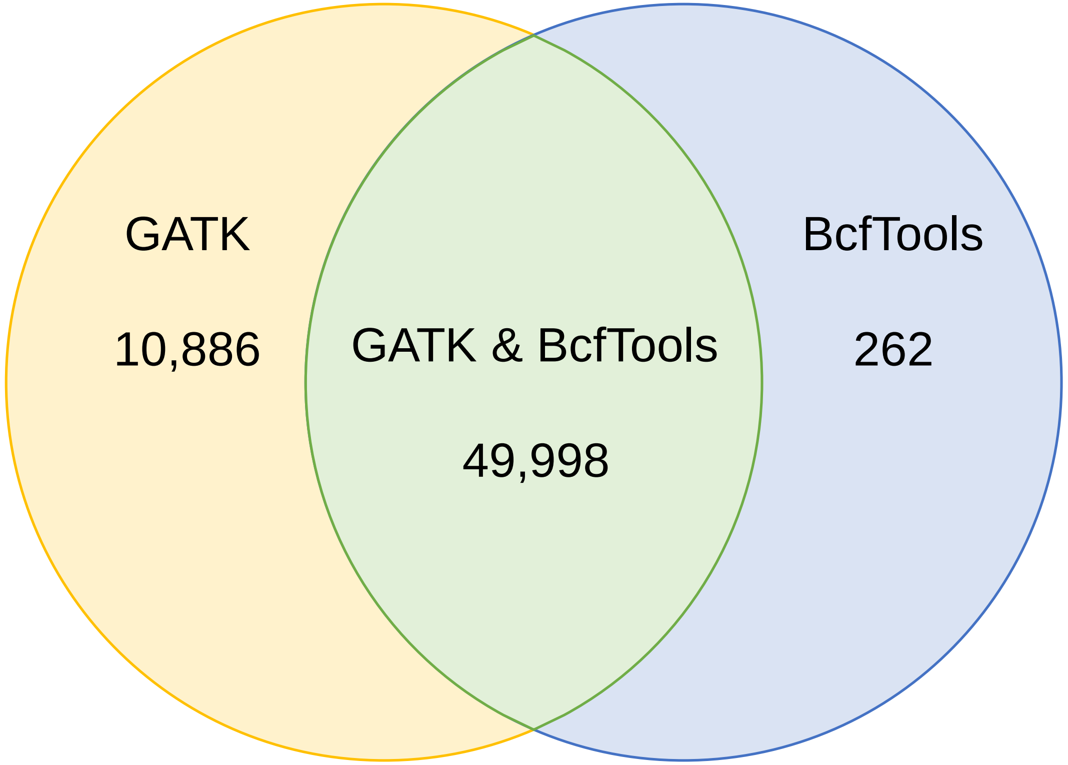

Supplementary Figure 2. Mutations detected by the variant calling programs GATK and BcfTools

Venn diagram of the mutations detected by GATK and BcfTools. Most mutations were detected by both programs, but some mutations were detected by only one of the programs.

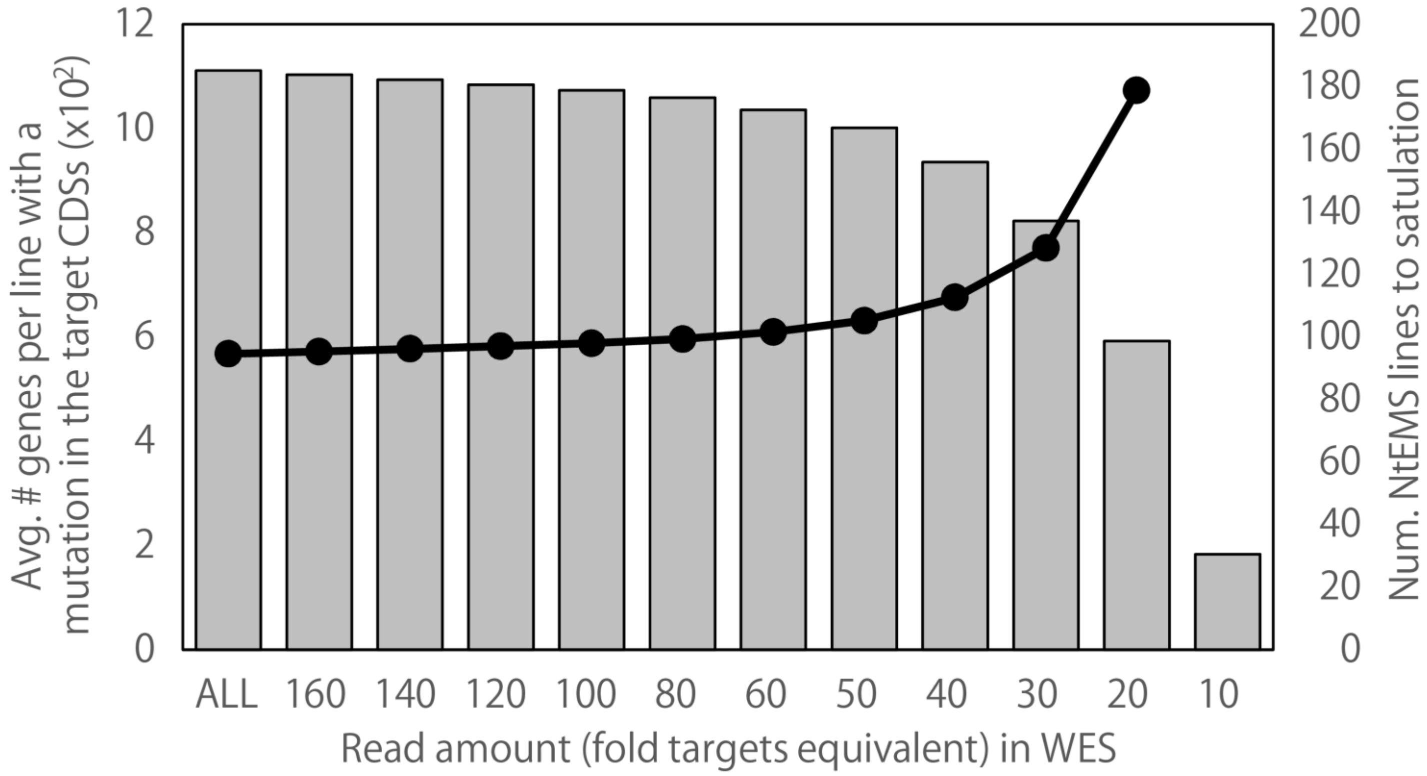

Supplementary Figure 3. The number of possible alternated genes by read amount and the population size required for saturation mutagenesis
Bar graph shows the average numbers of genes (per line) with at least one mutation within their CDS regions. The number of NtEMS lines required to achieve saturation mutagenesis (right axis) was also shown based on the probability formula by Clarke and Carbon (1976).

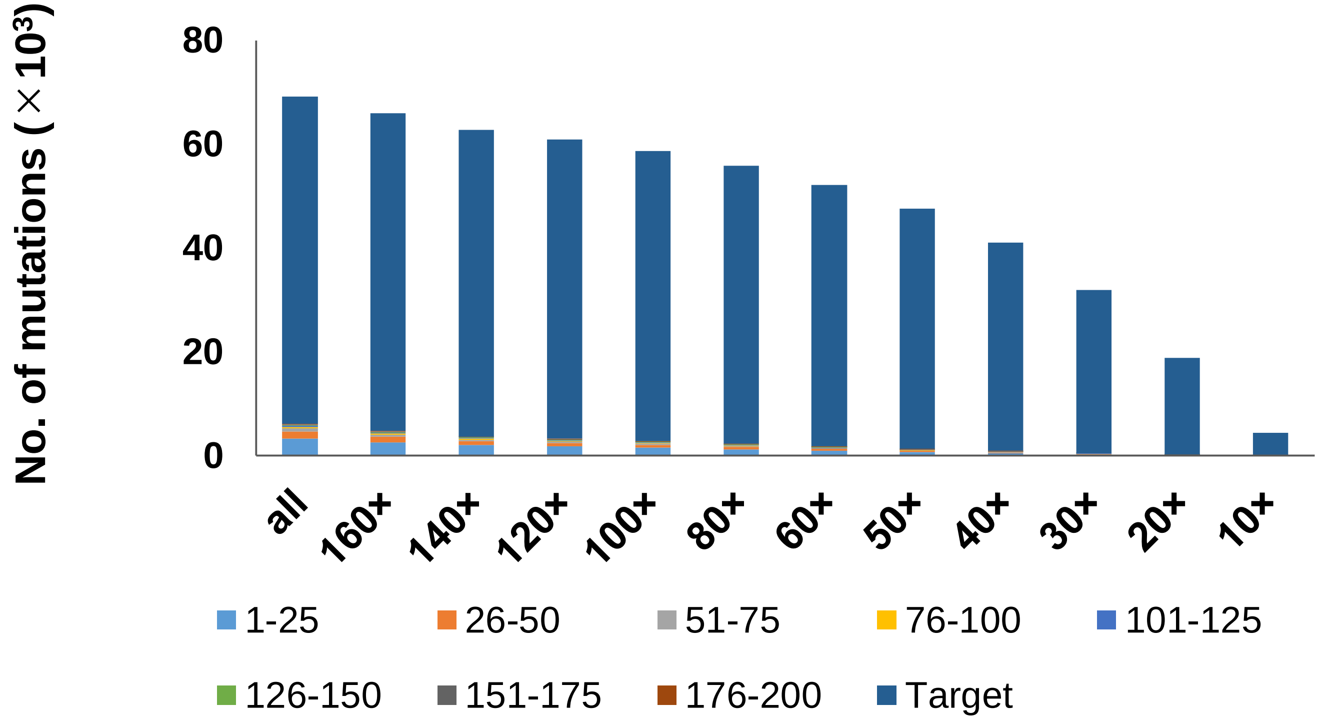

Supplementary Figure 4. Stacked bar chart of mutations detected in the target and the flanking regions for different amount of sequence reads.

The data are the total number of mutations detected in the 19 NtEMS lines with different numbers of sequencing reads: all clean sequencing reads ("all") and down-sampled sequencing reads (160×, 140×, 120×, 100×, 80×, 60×, 50×, 40×, 30×, 20×, and 10× total CDS length equivalent) by whole-exome sequencing. More mutations were detected in the target regions than in the flanking regions 1–25 to 176–200 distant from the target regions.
