# Supplementary material for: Highly Efficient and Comprehensive Identification of Ethyl Methanesulfonate-Induced Mutations in Nicotiana tabacum L. by Whole-Genome and Whole-Exome Sequencing
Source: Front Plant Sci. 2021 Jun 1;12:671598. doi: 10.3389/fpls.2021.671598 (PMC8204250; doi:10.3389/fpls.2021.671598)
Supplement: Supplementary Figure 1 — Target coverage and identity in the Nitab-v4.5_wes sequences. Histograms of the BLAST mapping of the target regions to Nitab-v4.5_wes sequences. (A) Percentage of query length; (B) Percentage identity. Most of the target regions had very high (>90%) coverage and identity. [file Data_Sheet_1.zip › Supplementary materials/Supplementary Table 10.docx]

**Supplementary Table 10** Whole-genome sequencing (WGS) statistics for 19 NtEMS lines and a technical replicate

|  | NtEMS-01 | NtEMS-02 | NtEMS-03 | NtEMS-04 | NtEMS-05 |
| --- | --- | --- | --- | --- | --- |
| Total read bases | 253,059,989,470 | 249,836,592,168 | 219,033,741,278 | 251,933,235,624 | 258,548,535,960 |
| Fold genome | 60.83 | 60.05 | 52.65 | 60.56 | 62.15 |
| Post-filtering bases | 224,781,032,761 | 224,724,363,518 | 201,098,241,987 | 226,448,145,845 | 229,074,964,344 |
| Total bases aligned | 215,468,131,166 | 214,884,372,429 | 192,596,690,473 | 217,012,123,286 | 219,767,697,030 |
| Bases aligned (%) | 95.86 | 95.62 | 95.77 | 95.83 | 95.94 |
| On bait bases | 4,485,899,065 | 4,540,922,762 | 4,024,386,415 | 4,557,403,899 | 4,629,420,650 |
| Near bait bases | 4,282,391,564 | 4,355,494,495 | 3,861,492,435 | 4,360,068,778 | 4,445,664,569 |
| % on/near bait | 4.07 | 4.14 | 4.09 | 4.11 | 4.13 |
| Mean target CDS coverage | 83.44 | 77.45 | 73.69 | 78.14 | 81.60 |
| % target CDS, read at ≥ 1× | 99.84 | 99.84 | 99.84 | 99.84 | 99.84 |
| % target CDS, read at ≥ 10× | 99.79 | 99.79 | 99.78 | 99.79 | 99.80 |
| % target CDS, read at ≥ 20× | 99.28 | 99.40 | 98.74 | 99.28 | 99.48 |
| % target CDS, read at ≥ 30× | 95.69 | 96.33 | 94.46 | 95.31 | 96.64 |
| % target CDS, read at ≥ 50× | 65.25 | 68.94 | 42.16 | 70.77 | 72.52 |

Supplementary Table 10 (continued)

|  | NtEMS-06 | NtEMS-07 | NtEMS-08 | NtEMS-09 | NtEMS-10 |
| --- | --- | --- | --- | --- | --- |
| Total read bases | 251,452,160,194 | 257,671,490,814 | 254,164,791,238 | 276,196,341,444 | 243,192,623,878 |
| Fold genome | 60.44 | 61.93 | 61.09 | 66.39 | 58.45 |
| Post-filtering bases | 223,540,538,769 | 227,521,889,178 | 226,351,209,281 | 244,445,884,165 | 223,352,833,991 |
| Total bases aligned | 214,560,110,756 | 218,548,439,049 | 217,081,571,334 | 235,248,201,881 | 214,993,526,189 |
| Bases aligned (%) | 95.98 | 96.06 | 95.90 | 96.24 | 96.26 |
| On bait bases | 4,511,858,498 | 4,568,265,513 | 4,593,842,979 | 4,872,785,233 | 4,500,890,806 |
| Near bait bases | 4,331,438,728 | 4,379,315,507 | 4,412,266,244 | 4,654,804,933 | 4,322,077,889 |
| % on/near bait | 4.12 | 4.09 | 4.15 | 4.05 | 4.10 |
| Mean target CDS coverage | 81.26 | 86.14 | 80.09 | 80.85 | 76.20 |
| % target CDS, read at ≥ 1× | 99.83 | 99.83 | 99.84 | 99.84 | 99.84 |
| % target CDS, read at ≥ 10× | 99.71 | 99.60 | 99.80 | 99.80 | 99.79 |
| % target CDS, read at ≥ 20× | 99.25 | 99.22 | 99.45 | 99.60 | 99.34 |
| % target CDS, read at ≥ 30× | 96.11 | 96.19 | 96.53 | 97.40 | 96.11 |
| % target CDS, read at ≥ 50× | 67.70 | 68.66 | 71.15 | 82.43 | 68.92 |

Supplementary Table 10 (continued)

|  | NtEMS-11 | NtEMS-12 | NtEMS-13 | NtEMS-14 | NtEMS-15 |
| --- | --- | --- | --- | --- | --- |
| Total read bases | 274,637,498,078 | 241,535,802,048 | 246,772,980,650 | 253,893,489,236 | 252,796,992,770 |
| Fold genome | 66.01 | 58.06 | 59.32 | 61.03 | 60.76 |
| Post-filtering bases | 239,176,835,805 | 213,105,757,306 | 220,205,473,947 | 221,995,941,045 | 227,102,662,291 |
| Total bases aligned | 229,639,886,989 | 205,158,296,871 | 212,052,204,961 | 213,318,048,049 | 218,594,039,280 |
| Bases aligned (%) | 96.01 | 96.27 | 96.30 | 96.09 | 96.25 |
| On bait bases | 4,711,085,987 | 4,298,853,003 | 4,439,356,078 | 4,429,778,417 | 4,575,343,761 |
| Near bait bases | 4,490,028,936 | 4,120,097,590 | 4,252,350,696 | 4,224,288,679 | 4,387,529,340 |
| % on/near bait | 4.01 | 4.10 | 4.10 | 4.06 | 4.10 |
| Mean target CDS coverage | 90.38 | 74.34 | 75.69 | 84.55 | 75.78 |
| % target CDS, read at ≥ 1× | 99.83 | 99.84 | 99.84 | 99.84 | 99.84 |
| % target CDS, read at ≥ 10× | 99.75 | 99.78 | 99.79 | 99.79 | 99.80 |
| % target CDS, read at ≥ 20× | 99.39 | 99.18 | 99.36 | 99.28 | 99.46 |
| % target CDS, read at ≥ 30× | 96.38 | 95.64 | 96.17 | 95.91 | 96.55 |
| % target CDS, read at ≥ 50× | 74.18 | 58.34 | 65.73 | 62.44 | 72.49 |

Supplementary Table 10 (continued)

|  | NtEMS-16 | NtEMS-17 | NtEMS-18 | NtEMS-19 | NtEMS-19-rep2 |
| --- | --- | --- | --- | --- | --- |
| Total read bases | 245,145,027,000 | 269,588,315,684 | 252,549,353,072 | 251,773,470,376 | 246,347,672,842 |
| Fold genome | 58.92 | 64.80 | 60.70 | 60.52 | 59.21 |
| Post-filtering bases | 220,789,068,260 | 237,786,288,130 | 225,747,481,987 | 224,296,195,166 | 221,655,797,841 |
| Total bases aligned | 212,101,511,734 | 228,183,568,527 | 216,981,063,295 | 215,636,084,453 | 213,052,777,833 |
| Bases aligned (%) | 96.07 | 95.96 | 96.12 | 96.14 | 96.12 |
| On bait bases | 4,458,563,222 | 4,730,636,986 | 4,572,374,215 | 4,540,828,549 | 4,487,947,478 |
| Near bait bases | 4,292,832,035 | 4,524,085,550 | 4,395,344,435 | 4,371,235,107 | 4,316,547,668 |
| % on/near bait | 4.13 | 4.06 | 4.13 | 4.13 | 4.13 |
| Mean target CDS coverage | 74.63 | 82.32 | 75.79 | 74.66 | 79.22 |
| % target CDS, read at ≥ 1× | 99.79 | 99.84 | 99.84 | 99.84 | 99.84 |
| % target CDS, read at ≥ 10× | 99.74 | 99.80 | 99.79 | 99.79 | 99.79 |
| % target CDS, read at ≥ 20× | 99.22 | 99.53 | 99.39 | 99.43 | 99.33 |
| % target CDS, read at ≥ 30× | 96.05 | 96.97 | 96.41 | 96.52 | 96.12 |
| % target CDS, read at ≥ 50× | 66.71 | 77.06 | 71.53 | 70.67 | 66.77 |
