# Supplementary material for: Highly Efficient and Comprehensive Identification of Ethyl Methanesulfonate-Induced Mutations in Nicotiana tabacum L. by Whole-Genome and Whole-Exome Sequencing
Source: Front Plant Sci. 2021 Jun 1;12:671598. doi: 10.3389/fpls.2021.671598 (PMC8204250; doi:10.3389/fpls.2021.671598)
Supplement: Supplementary Figure 1 — Target coverage and identity in the Nitab-v4.5_wes sequences. Histograms of the BLAST mapping of the target regions to Nitab-v4.5_wes sequences. (A) Percentage of query length; (B) Percentage identity. Most of the target regions had very high (>90%) coverage and identity. [file Data_Sheet_1.zip › Supplementary materials/Supplementary Table 5.docx]

**Supplementary Table 5** Whole-exome sequencing (WES) statistics for 19 NtEMS lines and a technical replicate

|  | NtEMS-01 | NtEMS-02 | NtEMS-03 | NtEMS-04 | NtEMS-05 |
| --- | --- | --- | --- | --- | --- |
| EMS conc. (%) | 0.8 | 0.8 | 0.6 | 0.6 | 0.8 |
| Total read bases | 15,615,276,498 | 13,776,378,992 | 12,871,779,360 | 12,357,423,528 | 12,760,282,228 |
| Fold target | 223.30 | 197.00 | 184.07 | 176.71 | 182.47 |
| Post-filtering bases | 12,086,913,410 | 11,291,893,122 | 10,748,075,186 | 10,435,488,266 | 10,800,300,367 |
| Total bases aligned | 11,879,839,425 | 11,102,459,458 | 10,575,987,577 | 10,264,819,542 | 10,621,318,695 |
| Bases aligned (%) | 98.29 | 98.32 | 98.40 | 98.36 | 98.34 |
| On bait bases | 8,445,130,836 | 7,883,260,275 | 7,468,462,294 | 7,313,951,227 | 7,600,879,338 |
| Near bait bases | 639,577,137 | 610,601,453 | 576,776,936 | 546,055,700 | 557,667,833 |
| % on/near bait | 76.47 | 76.50 | 76.07 | 76.57 | 76.81 |
| Mean target CDS coverage | 97.16 | 90.83 | 86.79 | 83.90 | 87.49 |
| % target CDS, read at ≥ 1× | 99.45 | 99.45 | 99.44 | 99.42 | 99.43 |
| % target CDS, read at ≥ 10× | 98.78 | 98.75 | 98.71 | 98.67 | 98.69 |
| % target CDS, read at ≥ 20× | 98.22 | 98.14 | 98.05 | 97.95 | 98.02 |
| % target CDS, read at ≥ 30× | 97.46 | 97.26 | 97.01 | 96.79 | 97.01 |
| % target CDS, read at ≥ 50× | 94.47 | 93.69 | 92.69 | 91.99 | 92.81 |

Supplementary Table 5 (continued)

|  | NtEMS-06 | NtEMS-07 | NtEMS-08 | NtEMS-09 | NtEMS-10 |
| --- | --- | --- | --- | --- | --- |
| EMS conc. (%) | 0.6 | 0.8 | 0.6 | 0.6 | 0.8 |
| Total read bases | 11,561,155,486 | 13,596,821,394 | 13,057,109,512 | 15,074,860,444 | 11,951,493,216 |
| Fold target | 165.32 | 194.43 | 186.72 | 215.57 | 170.91 |
| Post-filtering bases | 10,960,476,065 | 12,860,279,399 | 10,399,562,970 | 12,905,867,163 | 10,293,559,531 |
| Total bases aligned | 10,795,402,127 | 12,647,469,218 | 10,221,336,966 | 12,697,942,053 | 10,117,483,689 |
| Bases aligned (%) | 98.49 | 98.35 | 98.29 | 98.39 | 98.29 |
| On bait bases | 7,847,413,322 | 8,928,912,791 | 7,395,616,991 | 9,205,189,739 | 7,212,349,049 |
| Near bait bases | 556,828,205 | 541,428,700 | 513,470,876 | 660,076,017 | 536,214,530 |
| % on/near bait | 77.85 | 74.88 | 77.38 | 77.69 | 76.59 |
| Mean target CDS coverage | 92.92 | 102.90 | 84.27 | 106.95 | 83.44 |
| % target CDS, read at ≥ 1× | 99.46 | 99.47 | 99.39 | 99.45 | 99.44 |
| % target CDS, read at ≥ 10× | 98.61 | 98.51 | 98.63 | 98.78 | 98.67 |
| % target CDS, read at ≥ 20× | 97.80 | 97.80 | 97.93 | 98.26 | 97.95 |
| % target CDS, read at ≥ 30× | 96.58 | 96.86 | 96.84 | 97.57 | 96.79 |
| % target CDS, read at ≥ 50× | 92.04 | 93.44 | 92.35 | 95.02 | 91.92 |

Supplementary Table 5 (continued)

|  | NtEMS-11 | NtEMS-12 | NtEMS-13 | NtEMS-14 | NtEMS-15 |
| --- | --- | --- | --- | --- | --- |
| EMS conc. (%) | 0.8 | 0.8 | 0.8 | 0.8 | 0.6 |
| Total read bases | 13,803,214,692 | 13,424,308,546 | 14,089,789,466 | 12,575,174,882 | 11,926,103,230 |
| Fold target | 197.39 | 191.97 | 201.48 | 179.82 | 170.54 |
| Post-filtering bases | 11,677,439,412 | 11,512,902,534 | 12,336,074,350 | 10,855,831,278 | 9,896,393,999 |
| Total bases aligned | 11,488,555,562 | 11,318,226,390 | 12,132,825,534 | 10,666,785,547 | 9,716,796,937 |
| Bases aligned (%) | 98.38 | 98.31 | 98.35 | 98.26 | 98.19 |
| On bait bases | 8,230,470,915 | 8,049,287,502 | 8,612,399,795 | 7,588,167,590 | 6,882,147,804 |
| Near bait bases | 629,615,988 | 564,458,117 | 634,856,733 | 608,816,812 | 504,286,613 |
| % on/near bait | 77.12 | 76.11 | 76.22 | 76.85 | 76.02 |
| Mean target CDS coverage | 95.78 | 92.59 | 100.66 | 87.40 | 78.27 |
| % target CDS, read at ≥ 1× | 99.43 | 99.42 | 99.47 | 99.46 | 99.42 |
| % target CDS, read at ≥ 10× | 98.70 | 98.69 | 98.76 | 98.72 | 98.64 |
| % target CDS, read at ≥ 20× | 98.09 | 98.06 | 98.18 | 98.05 | 97.88 |
| % target CDS, read at ≥ 30× | 97.19 | 97.12 | 97.35 | 97.04 | 96.66 |
| % target CDS, read at ≥ 50× | 93.45 | 93.42 | 94.20 | 92.83 | 91.42 |

Supplementary Table 5 (continued)

|  | NtEMS-16 | NtEMS-17 | NtEMS-18 | NtEMS-19 | NtEMS-19-rep2 |
| --- | --- | --- | --- | --- | --- |
| EMS conc. (%) | 0.6 | 0.8 | 0.8 | 0.8 | 0.8 |
| Total read bases | 14,959,157,268 | 12,623,008,280 | 17,090,548,350 | 12,866,744,308 | 18,938,654,632 |
| Fold target | 213.91 | 180.51 | 244.39 | 183.99 | 270.82 |
| Post-filtering bases | 12,177,017,025 | 11,013,499,853 | 13,448,482,391 | 10,583,849,085 | 15,247,034,134 |
| Total bases aligned | 11,957,175,415 | 10,829,316,790 | 13,198,338,018 | 10,392,692,684 | 14,982,653,132 |
| Bases aligned (%) | 98.19 | 98.33 | 98.14 | 98.19 | 98.27 |
| On bait bases | 8,577,135,177 | 7,691,607,496 | 9,370,849,532 | 7,437,613,253 | 10,638,189,647 |
| Near bait bases | 629,062,365 | 562,321,895 | 672,595,375 | 530,212,509 | 794,982,164 |
| % on/near bait | 98.30 | 89.82 | 106.34 | 84.81 | 123.42 |
| Mean target CDS coverage | 93.35 | 93.40 | 93.41 | 93.36 | 93.48 |
| % target CDS, read at ≥ 1× | 99.40 | 99.45 | 99.46 | 99.41 | 99.51 |
| % target CDS, read at ≥ 10× | 98.72 | 98.69 | 98.80 | 98.66 | 98.87 |
| % target CDS, read at ≥ 20× | 98.17 | 98.01 | 98.32 | 98.00 | 98.45 |
| % target CDS, read at ≥ 30× | 97.43 | 96.96 | 97.73 | 96.97 | 97.97 |
| % target CDS, read at ≥ 50× | 94.58 | 92.70 | 95.58 | 92.75 | 96.34 |
